# Prehospital Validation of the Scale to Identify Large Vessel Occlusion in Suspected Stroke Patients

**DOI:** 10.1159/ced/accag004

**Published:** 2026-08-10

**Authors:** Takahiro Shimizu, Yasuhiro Hasegawa, Yoshifumi Tsuboi, Toshihiro Ueda, Jinichi Sasanuma, Atsushi Tsuchiya, Ririko Takeda, Yohichi Imaizumi, Hajime Ono, Satoshi Inoue, Hiroyuki Nozaki, Takehiko Nagao, Hajime Nakanishi, Shojiro Kawaguchi, Kengo Yokomine, Hisanao Akiyama, Yoshihisa Yamano, on behalf of the Kawasaki Stroke Network Investigators

**Affiliations:** a Department of Neurology, St. Marianna University School of Medicine, Kawasaki, Japan; b Stroke Center, Shin-Yurigaoka General Hospital, Kawasaki, Japan; c Department of Neurosurgery, Kawasaki Saiwai Hospital, Kawasaki, Japan; d Department of Neuroendovascular Therapy, St. Marianna University School of Medicine, Kawasaki, Japan; e Department of Neurosurgery, Shin-Yurigaoka General Hospital, Kawasaki, Japan; f Department of Neurology, Kanto Rosai Hospital, Kawasaki, Japan; g Department of Neurosurgery, Teikyo University School of Medicine, University Hospital Mizonokuchi, Kawasaki, Japan; h Department of Neurosurgery, AOI Universal Hospital, Kawasaki, Japan; i Department of Neurology, Municipal Tama Hospital, Kawasaki, Japan; j Department of Neurology, Kawasaki Municipal Hospital, Kawasaki, Japan; k Department of Neurology, Nippon Medical School Musashikosugi Hospital, Kawasaki, Japan; l Department of Neurosurgery, Ohta General Hospital, Kawasaki, Japan; m Department of Neurosurgery, Asao General Hospital, Kawasaki, Japan; n Department of Neurosurgery, Daiichi Hospital, Kawasaki, Japan

**Keywords:** Prehospital stroke scale, Emergency medical service, Stroke, Large vessel occlusion, Mechanical thrombectomy

## Abstract

**Introduction:**

Timely identification and transfer of patients with large vessel occlusion (LVO) to thrombectomy-capable stroke centers (TSCs) for endovascular thrombectomy (EVT) remains a major challenge in prehospital stroke care. This study evaluated the clinimetric performance of the Cincinnati Prehospital Stroke Scale (CPSS), the Maria Prehospital Stroke Scale (MPSS) and novel LVO screening scales created by integrating four LVO-related items into these established prehospital assessments.

**Methods:**

Using prehospital electronic records linked with hospital data across Kawasaki City from April 2023 to March 2024, we retrospectively analyzed whether the addition of aphasia, unilateral spatial neglect, conjugate eye deviation, and atrial fibrillation (via ambulance ECG) improved prehospital LVO detection. These items were incorporated into the MPSS (MPSS+4 scale) and CPSS (CPSS+4 scale), and the predictive performance of four tools: CPSS, CPSS+4 scale, MPSS, and MPSS+4 scale was compared.

**Results:**

Among 1,653 transported patients, 207 (12.5%) had LVO. MPSS+4 scale demonstrated the highest predictive performance for LVO (AUROC 0.768). The addition of the four LVO-related items significantly improved LVO prediction for both MPSS and CPSS (*p* < 0.001 for each). Higher MPSS+4 scale scores were strongly and progressively associated with the likelihood of receiving EVT after hospital arrival. Adjusted odds ratios increased from 2.97 to 100.59 for scores 2–9 compared with a score of 1.

**Conclusion:**

Incorporating LVO-related items into prehospital stroke assessment substantially improved diagnostic precision for LVO, with MPSS+4 scale outperforming all other tools. The strong association between MPSS+4 scale scores and subsequent EVT implementation suggests that MPSS+4 scale may serve as a practical, clinically meaningful instrument for emergency medical technicians in prehospital triage of suspected acute stroke and identification of EVT candidates.

## Introduction

Current evidence indicates that endovascular thrombectomy (EVT) offers clinical benefit in appropriately selected patients with acute ischemic stroke (AIS), provided it is performed within a time-sensitive therapeutic window [1–3]. The efficacy of reperfusion therapy is known to decline with increasing time from stroke onset to the initiation of EVT (“onset-to-puncture time”) [4, 5]. A one-hour delay in reperfusion has been reported to result in a 19% decrease in the proportion of patients achieving favorable outcomes at 90 days [4]. While intravenous thrombolysis (IVT) is available around the clock at primary stroke centers (PSCs), EVT requires specialized facilities and expertise, and is therefore not universally accessible. Consequently, patients with AIS due to large vessel occlusion (LVO) must be promptly transferred to a thrombectomy-capable stroke center (TSC) to receive definitive care. However, this inevitably prolongs the time from symptom onset to EVT initiation, potentially reducing the expected therapeutic benefit. To address this issue, it is critical to accurately identify LVO cases during the prehospital phase using assessments conducted by emergency medical technicians (EMTs), thereby enabling direct transport of patients to TSCs.

Various prehospital LVO assessment tools intended for use by EMTs have been proposed, however, these studies often lack uniformity, with discrepancies in assessment items across studies, retrospective study designs [6, 7], and inclusion of evaluations conducted by physicians after hospital arrival rather than by emergency personnel on the scene [6, 8–13]. As a result, meta-analyses failed to demonstrate the most reliable prehospital method for diagnosing LVO [14, 15].

Standardized prehospital stroke assessment, transport decision-making, and treatment implementation are critically important for optimizing acute stroke care. To this end, the Japan Stroke Society has launched a project to standardize LVO scales. In this initiative, four additional items – aphasia, unilateral spatial neglect (USN), conjugate eye deviation, and arrhythmia – were prospectively evaluated alongside the Cincinnati Prehospital Stroke Scale (CPSS), a widely used prehospital stroke assessment tool developed prior to the advent of mechanical thrombectomy (MT), comprising facial droop, arm drift, and dysarthria [16]. Based on the findings of this project, the Society has formally requested the Fire and Disaster Management Agency of the Ministry of Internal Affairs and Communications to allow flexible implementation of LVO scales incorporating these items, tailored to local medical resources.

Recently, a systematic review and meta-analysis demonstrated that numerical CPSS score, with a threshold set at ≥2, may also be useful for identifying LVO [17], implying that use of the simpler CPSS may enhance compliance with the utilization of LVO screening instruments while maintaining the accuracy of prehospital LVO determination [18]. However, evidence remains insufficient to resolve the reciprocal challenge of balancing simplicity of assessment with diagnostic accuracy for LVO.

The MPSS, a modified version of the CPSS, has been developed as a prehospital stroke scale for EMTs to predict the likelihood of the performance of thrombolytic therapy after transportation (Table 1) [19]. In Kawasaki City (population of 1,557,683; area 142.7 km^2^), a citywide transportation protocol for stroke utilizing the MPSS was launched in January 2009 to facilitate timely access to IVT. Quality assurance monitoring has been conducted biannually since April 2009 [20, 21]. Since April 2023, evaluations for LVO have been implemented in parallel with the MPSS, and efforts have been initiated to collect baseline data on LVO-related transfers. This study aims to evaluate whether incorporating four additional items – aphasia, USN, conjugate eye deviation, and arrhythmia – as recommended by the Japan Stroke Society into the MPSS assessment improves the accuracy of LVO prediction, and to compare the predictive performance of the MPSS+4 scale (Table 1) with that of the CPSS based on the current dataset.

**Table 1.** The MPSS (Maria Prehospital Stroke Scale) score and four items added to the MPSS score to evaluate LVO

Facial droop 0 Normal 1 Abnormal	(The patient shows teeth or smiles) Both sides of face move equally One side of face does not move as well as the other	​
Arm drift 0 Normal 1 Abnormal, not severe 2 Abnormal, severe	(The patient closes the eyes and extends both arms straight out with palms-up for 10 s) Both arms raised equally One arm drifts down or pronates Only one arm raised or cannot complete request at all	​
Speech 0 Normal 1 Abnormal, not severe 2 Abnormal, severe	(Test this item by free talking or ordering to repeat a short phrase.) The patient can speak without slurring of words Slurred speech Not understandable speech or Unable to speak	​
Total	​	/5
Four items for the LVO triage	​	​	
Aphasia 0 Normal 1 Abnormal	(Show glasses or watch and ask “What is this?”) Correct answer Unable to answer	​	
USN 0 Normal 1 Abnormal	(Show four fingers and ask “How many fingers?”) Correct answer Wrong answer	​	
Conjugate eye deviation 0 Normal 1 deviation (+)	(To observe whether the conjugate eye deviation is present) Not present Present	​	
Atrial Fibrillation 0 None 1 AF detected on ECG	ECG assessment in the ambulance AF is not present AF is present	​	
Total	​	/4	

## Methods

In Kawasaki City, hospitals capable of administering IVT collaborated with Kawasaki City Fire Department to establish the Kawasaki Stroke Network (KSN). Since its inception in 2007, the MPSS has been utilized as the prehospital stroke triage tool within this network. In April 2022, the Kawasaki City Medical Control Council approved the addition of four LVO-related items recommended by the Japan Stroke Society – aphasia, USN, conjugate eye deviation, and atrial fibrillation (identified via ambulance ECG) – to the prehospital assessment protocol conducted by EMTs. This decision marked the establishment of a new transport system designed to facilitate EVT for patients with suspected LVO. In collaboration with Kawasaki City Fire Department, we developed standardized video-based training materials to improve EMT assessments of four newly introduced LVO-related items. Following the implementation of this training program for all EMTs, evaluation of the additional items commenced on April 1, 2023, alongside the existing MPSS assessment. Baseline data collection was initiated and will continue for 1 year to inform the development of a revised transfer protocol for patients with suspected LVO.

From April 1, 2023, to March 31, 2024, all patients transported to the network-affiliated hospitals were included in the analysis. As of April 2024, twelve institutions participate in the Kawasaki Stroke Network. Among them, seven facilities do not routinely perform EVT (PSC), while the remaining five hospitals provide EVT services on a 24/7 basis (TSC). Although EMTs performed assessments of the LVO-related items, they did not communicate the results of these assessments to the receiving hospitals, and were not used to determine hospital destination.

All patients underwent computed tomography or magnetic resonance imaging. Presence of LVO was evaluated on admission using magnetic resonance angiography or CT angiography at the time of hospital arrival. In this study, LVO was defined as an occlusion site affecting the internal carotid artery, the M1 or M2 segment of the middle cerebral artery, or the basilar artery.

The diagnostic accuracy for stroke and that for LVO were calculated using four different assessment strategies: (1) MPSS score only, (2) CPSS score only, (3) MPSS score plus four LVO-related items (MPSS+4 scale), and (4) CPSS score plus four LVO-related items (CPSS+4 scale). For each method, the area under the ROC curve (AUROC), accuracy, sensitivity, specificity, positive predictive value (PPV), and negative predictive value (NPV) at each cutoff point were computed.

The NIHSS score [22] was recorded at hospital arrival, 24 h after IVT and/or EVT, and 1 month after onset or at discharge in patients with ischemic stroke who received IVT or EVT. The Rankin Scale score before onset and the modified Rankin Scale (mRS) score [23] at 1 month after onset or at discharge were recorded. The mRS score at 3 months after onset was not obtained because the present study was carried out as quality assurance monitoring while adhering to fire department regulations in Japan.

All the MPSS-based transportation data are recorded in Microsoft Excel format (Microsoft Corporation, Tokyo, Japan) by Kawasaki City Fire Department. Clinical data for patients transported to each hospital are subsequently appended as anonymized, linkable datasets, in compliance with the Japanese Fire Service Act. Following unlinkable anonymization, the dataset is subjected to biannual statistical analysis for quality assurance monitoring by the Department of Neurology at St. Marianna University School of Medicine, which serves as the administrative office of the Kawasaki Stroke Network. Due to the anonymous nature of the data, informed consent was waived. In accordance with these regulations, interventional studies or follow-up investigations beyond the acute phase were not permitted. This study was approved by the St. Marianna University Bioethics Committee (#2509).

### Clinimetric Characteristics of the MPSS

The clinimetric characteristics of the MPSS have been described in detail elsewhere [19–21]. Briefly, the MPSS was developed by modifying the CPSS. The CPSS assesses three domains – facial droop, arm drift, and speech disturbance – each rated as either normal (0) or abnormal (1) by EMTs. A score of 1 or higher is considered indicative of stroke. In contrast, the MPSS incorporates a severity grading system. Facial droop is evaluated as normal (0) or abnormal (1), while arm drift and dysarthria are each rated on a three-point scale: normal (0), not severe (1), or severe (2). Consequently, the total MPSS score ranges from 0 to 5 (Table 1), representing an extension of the CPSS framework with added granularity for symptom severity. Operationally, an MPSS score ≥1 is considered equivalent to CPSS ≥1 in identifying suspected stroke.

Notably, MPSS scores assigned by EMTs show a strong positive correlation with National Institutes of Health Stroke Scale (NIHSS) scores assessed concurrently by physicians in the emergency department (Spearman’s rho = 0.89, *p* < 0.001). Furthermore, the proportion of patients receiving IVT increases progressively with higher MPSS scores: 0% (score 0), 4.1% (score 1), 8.8% (score 2), 13.0% (score 3), 20.3% (score 4), and 31.5% (score 5). The diagnostic accuracy of EMTs using MPSS for stroke identification was 0.737 (95% confidence interval [CI], 0.688–0.786), while the predictive accuracy for subsequent administration of iv-tPA was 0.689 (95% CI, 0.645–0.732) [19–21].

### Evaluation of LVO-Related Four Items

Aphasia is assessed through naming tasks (e.g., showing a watch or a pair of eye glasses). USN is evaluated using the “four-finger method” as recommended by the Japan Stroke Society, whereby EMTs hold up four fingers in front of the patient and ask, “How many fingers am I holding up?” [24]. Conjugate eye deviation is determined by observing the patient’s eye position. Atrial fibrillation is diagnosed via ECG examination after the patient has been placed in the ambulance. These LVO-related four items are rated as either normal (0) or abnormal (1) by EMTs (Table 1).

### Statistical Analysis

The unpaired Student’s *t* test was used to compare continuous variables, and Pearson’s χ^2^ test was used for nominal parameters. The Mann-Whitney U test was used for data that were not normally distributed. Logistic regression analysis was performed to identify predictors of stroke and those of LVO. For this purpose, univariate analyses were conducted on age, male sex, MPSS score, aphasia, USN (four-finger method), conjugate eye deviation, and presence of atrial fibrillation. Variables with *p* < 0.1 in univariate analysis were then included in a multivariate analysis.

Diagnostic accuracies of prehospital assessment tools by EMTs were tested by receiver operating characteristic (ROC) curve analysis and expressed as the area under the receiver operating characteristic curve (AUROC). Ideal discrimination produces AUROC of 1.0, and discrimination that is no better than chance produces an AUROC of 0.5. Discriminatory capacity was considered as poor (AUROC, 0.5–0.7), acceptable (AUROC ≥ 0.7), excellent (AUROC ≥ 0.8), or outstanding (AUROC ≥ 0.9). To determine the presence of statistically significant differences in the discriminative ability, the DeLong method was employed to the two ROC curves. The optimal cutoff value was determined using the Youden index. Sensitivity, specificity, PPV, NPV, and accuracy were calculated at the cutoff point, with 95% CIs computed using the Wilson score method.

Predictive value of the each MPSS+4 scale score for EVT after transportation was analyzed via multivariate logistic regression analysis. All statistical analyses were performed using IBM SPSS Statistics for Windows version 29 software (Chicago, IL, USA). All statistical tests were 2-sided, and probability values <0.05 were considered significant.

## Results

### Patient Characteristics

A total of 1,653 patients were transported to KSN-affiliated facilities based on MPSS assessment (940 males; mean age, 73.7 ± 14.8 years). Of these, 1,190 cases were diagnosed with stroke or transient ischemic attack (TIA), including 763 with cerebral infarction, 301 with intracerebral hemorrhage, 36 with subarachnoid hemorrhage, and 90 with TIA (Table 2). Prehospital assessments by EMTs identified aphasia in 309 (18.7%), USN by the four-finger test in 95 (5.7%), conjugate eye deviation in 278 (16.8%), and atrial fibrillation on ambulance ECG in 321 (19.4%). LVO was identified in 207 (12.5%) patients, and 172 ischemic stroke patients (22.5% of brain infarction) received intravenous thrombolysis (tPA) and/or MT. Among all transported cases, 463 (28.0%) were ultimately diagnosed as stroke mimics, with a wide variety of underlying conditions (online suppl. Table 1; for all online suppl. material, see https://doi.org/10.1159/ced/accag004). The most common diagnosis was epilepsy or seizure (16.2%), followed by subdural hematoma (9.5%); dizziness or vertigo (6.5%), syncope (6.3%), primary headache or migraine (3.9%), and hypoglycemia (3.7%).

**Table 2.** Patient characteristics

Total number of transferred patients, *n*	1653
Male sex, *n* (%)	940 (56.9)
Age, mean±SD, years	73.7±14.8
Prehospital evaluation by EMTs	​
Facial droop, *n* (%)	541 (32.7)
Arm drift, *n* (%)	994 (60.0)
Dysarthria, *n* (%)	1,044 (63.0)
Aphasia, *n* (%)	309 (18.7)
USN, *n* (%)	95 (5.7)
Conjugate eye deviation, *n* (%)	279 (16.8)
Atrial fibrillation, *n* (%)	321 (19.4)
Type of stroke center transferred by EMS	​
PSC, *n* (%)	610 (36.9)
TSC, *n* (%)	1,043 (63.1)
Detection-to-door time, median (min.–max.) min	24 (16–114)
Final diagnosis	​
Transient Ischemic Attacks	90 (5.4)
Brain infarction, *n* (%)	763 (46.2)
LVO, *n* (%)	207 (12.5)
ICA, *n* (%)	68 (4.8)
M1, *n* (%)	80 (4.8)
M2, *n* (%)	45 (2.7)
BA, *n* (%)	15 (0.9)
Brain hemorrhage, *n* (%)	301 (18.2)
Subarachnoid hemorrhage, *n* (%)	36 (2.2)
Stroke mimics, *n* (%)	463 (28.0)
Reperfusion therapy (iv-tPA and/or MT) in brain infarction	172 (22.5%)
iv-tPA only, *n* (%)	54 (7.1)
iv-tPA with MT, *n* (%)	46 (6.0%)
MT only, *n* (%)	72 (9.4%)

SD, standard deviation; EMS, emergency medical service; ICA, internal carotid artery; M1, horizontal portion of the middle cerebral artery; M2, insular portion of the middle cerebral artery; BA, basilar artery; iv-tPA, intravenous therapy using tissue plasminogen activator; MT, mechanical thrombectomy.

### Prediction of Stroke and LVO by Prehospital Assessment of EMTs

Univariate analyses identified age, facial droop, arm drift, dysarthria, aphasia, USN, conjugate eye deviation, and atrial fibrillation as significantly associated with LVO (see unadjusted ORs in Table 3). In multivariate models, the four additional cortical/arrhythmic items – aphasia, USN, conjugate eye deviation, and atrial fibrillation – remained independent predictors of LVO after adjustment for core MPSS items; the adjusted odds ratios and 95% CIs are presented in Table 3. For stroke (all etiologies), only the core motor/speech items (facial droop, arm drift, dysarthria) retained independent significance.

**Table 3.** Odds ratios and 95% CIs for prehospital assessment items evaluated by EMTs in predicting stroke and LVO

​	Prediction of stroke				Prediction of LVO			
Prehospital evaluation	unadjusted ORs	*p*	adjusted ORs	*p*	unadjusted ORs	*p*	adjusted ORs	*p*
Age	1.007 (1.000–1.014)	0.057	0.999 (0.991–1.007)	0.843	1.026 (1.015–1.037)	<0.001	1.023 (1.009–1.036)	<0.001
Male sex	1.168 (0.946–1.441)	0.148	–	​	0.922 (0.701–1.214)	0.565	–	​
MPSS score	​	​	​	​	​	​	​	​
Facial droop (0,1)	2.656 (2.069–3.408)	<0.001	1.572 (1.194–2.071)	0.001	1.944 (1.481–2.553)	<0.001	1.418 (1.009–1.992)	0.044
Arm drift (0,1,2)	2.304 (2.000–2.654)	<0.001	1.789 (1.528–2.095)	<0.001	2.323 (1.940–2.782)	<0.001	2.988 (1.766–5.057)	<0.001
Dysarthria (0,1,2)	2.156 (1.830–2.541)	<0.001	1.637 (1.341–1.998)	<0.001	2.363 (1.934–2.887)	<0.001	1.708 (1.036–2.818)	0.036
Aphasia	1.813 (1.353–2.430)	<0.001	0.770 (0.536–1.107)	0.159	2.830 (2.095–3.823)	<0.001	1.769 (1.230–2.544)	0.002
Unilateral Spatial Neglect	8.794 (3.548–21.795)	<0.001	5.307 (2.085–13.511)	0.369	3.116 (1.981–4.901)	<0.001	1.708 (1.011–2.888)	0.046
Conjugate eye deviation	3.119 (2.198–4.428)	<0.001	1.202 (0.804–1.798)	0.369	4.152 (3.071–5.614)	<0.001	2.260 (1.575–3.244)	<0.001
Atrial Fibrillation	2.034 (1.513–2.735)	<0.001	1.341 (0.966–1.861)	0.079	3.582 (2.668–4.807)	<0.001	1.917 (1.346–2.731)	<0.001

MPSS, Maria Prehospital Stroke scale; USN, unilateral spatial neglect evaluated by the four-finger method; Stroke, diagnosis of brain infarction, brain hemorrhage, or subarachnoid hemorrhage.

ROC analysis for stroke detection showed similar discrimination across the four tools, with AUROCs clustered around 0.7 and no significant pairwise differences (see Table 4A). By contrast, for LVO prediction the addition of the four items improved discrimination: the MPSS+4 scale achieved the highest AUROC (0.768), followed by CPSS+4 (0.738) and MPSS (0.736); the standalone CPSS did not exceed an AUROC of 0.7 (see Table 4B). Pairwise DeLong comparisons indicated that the improvements with the +4 versions were statistically significant (*p* < 0.05 for relevant comparisons); consult Table 4B for exact *p* values.

**Table 4.** AUROCs for predicting stroke and LVO in each prehospital assessment tool and pairwise comparisons

​	AUROC (95% CI)	MPSS+4 scale	CPSS+4 scale	MPSS	CPSS
A. Prediction of stroke	​	​	​	​	​
MPSS+4 scale	0.707 (0.680–0.733)	NA	​	​	​
CPSS+4 scale	0.702 (0.675–0.729)	0.148*	NA	​	​
MPSS score	0.706 (0.679–0.732)	0.712*	0.542*	NA	​
CPSS score	0.695 (0.668–0.722)	0.108*	0.281*	0.055*	NA
B. Prediction of LVO	​	​	​	​	​
MPSS+4 scale	0.768 (0.733–0.803)	NA	​	​	​
CPSS+4 scale	0.738 (0.704–0.771)	0.029*	NA	​	​
MPSS	0.736 (0.700–0.772)	<0.001*	0.042*	NA	​
CPSS	0.672 (0.638–0.706)	<0.001*	<0.001*	<0.001*	NA

Statistically significant values are shown in bold.

CI, confidence interval.

*p value in pairwise comparison with DeLong method.

Using the Youden index to define optimal cutoffs, MPSS showed the highest accuracy for overall stroke detection (accuracy 0.596), whereas MPSS+4 provided the best accuracy for LVO detection (accuracy 0.791). Detailed sensitivity, specificity, PPV, and NPV for each tool at their optimal cutoffs are reported in Table 5; these values illustrate that MPSS+4 achieves a more favorable balance between sensitivity and specificity for identifying LVO in the prehospital setting compared with the other scales. For reference, the sensitivity, specificity, PPV, and NPV for each MPSS+4 scale score at all possible cutoff points are provided in online supplementary Table 2.

**Table 5.** Predictive performance of assessment tools for detecting stroke and LVO at each cutoff points calculated by Youden index

Assessment tools (score range)	Cutoff point	Accuracy (95% CI)	Sensitivity, % (95% CI)	Specificity, % (95% CI)	PPV, % (95% CI)	NPV, % (95% CI)
Prediction of stroke	​	​	​	​	​	​
MPSS+4 scale (0–9 point)	≥3	0.559 (0.534–0.583)	41.5 (38.7–44.5)	86.4 (83.2–89.1)	86.7 (83.6–89.4)	41.0 (38.1–43.9)
CPSS+4 sale (0–7 point)	≥2	0.488 (0.464–0.513)	28.5 (25.9–31.3)	92.5 (89.9–94.5)	89.2 (85.5–92.0)	37.5 (34.9–40.2)
MPSS (0–5 point)	≥3	0.596 (0.572–0.620)	48.9 (46.0–51.9)	82.4 (78.8–85.4)	85.5 (82.6–88.1)	43.1 (40.0–46.2)
CPSS (0–3 point)	≥2	0.496 (0.472–0.520)	32.3 (27.6–33.0)	91.2 (88.4–93.3)	88.1 (84.4–91.0)	37.8 (35.1–40.5)
Prediction of LVO	​	​	​	​	​	​
MPSS+4 scale (0–9 point)	≥4	0.791 (0.770–0.810)	49.4 (43.1–55.6)	84.1 (82.1–85.9)	34.7 (29.9–39.9)	90.7 (89.0–92.1)
CPSS+4 scale (0–7 point)	≥3	0.780 (0.759–0.799)	49.4 (43.1–55.6)	82.9 (80.8–84.8)	33.1 (28.5–38.2)	90.5 (88.8–92.0)
MPSS (0–5 point)	≥3	0.773 (0.752–0.792)	46.5 (40.3–52.8)	82.5 (80.4–84.4)	31.2 (26.6–36.2)	90.0 (88.3–91.5)
CPSS (0–3 point)	≥2	0.733 (0.712–0.754)	38.6 (32.7–44.9)	79.3 (77.1–81.4)	24.3 (20.3–28.8)	88.3 (86.4–89.9)

Cutoff point was calculated by Youden index. Accuracy at cutoff point was calculated as (true positives + true negatives)/total number of patients. LVO was defined as an occlusion site affecting the internal carotid artery, the M1 or M2 segment of the middle cerebral artery, or the basilar artery. The Wilson score interval was used to calculate the 95% confidence interval.

MPSS, Maria Prehospital Stroke Scale; CPSS, Cincinnati prehospital stroke scale; PPV, positive predictive value; NPV, negative predictive value.

### Association between MPSS+4 Score and MT Implementation

When stratified by MPSS+4 score, there was a clear, monotonic relationship between higher prehospital scores and the likelihood of receiving MT after hospital arrival. In multivariate logistic regression using a score of 1 as the reference, adjusted odds ratios for MT increased progressively with each higher score, ranging from 2.97 to 100.59 for scores 2 through 9, respectively (see Table 6). This dose-response pattern supports the clinical relevance of the MPSS+4 score for anticipating MT candidacy in the field.

**Table 6.** Logistic regression analysis for the prediction of MT after transportation

​	​	Univariate	​	​	Multivariate	​
	OR	95% CI	*p*	OR	95% CI	*p*
Male sex	0.706	0.483–1.034	0.074	​	​	​
Age	1.013	0.999–1.028	0.070	​	​	​
MPSS+4 scale score	​	​	<0.001	​	​	<0.001
MPSS+4=1	1	reference	​	1	reference	​
MPSS+4=2	3.023	0.937–9.749	0.064	2.970	0.919–9.598	0.069
MPSS+4=3	5.185	1.699–15.826	0.004	5.029	1.644–15.386	0.005
MPSS+4=4	8.085	2.694–24.261	<0.001	7.898	2.625–23.764	<0.001
MPSS+4=5	17.466	6.022–50.661	<0.001	16.367	5.626–47.614	<0.001
MPSS+4=6	16.089	5.274–49.086	<0.001	15.765	5.146–48.300	<0.001
MPSS+4=7	33.787	10.830–105.407	<0.001	35.729	11.350–112.474	<0.001
MPSS+4=8	23.382	5.752–95.047	<0.001	24.892	6.026–102.828	<0.001
MPSS+4=9	79.500	4.194–1506.820	0.004	100.591	4.808–2104.482	0.003
Type of stroke center	​	​	<0.001	​	​	<0.001
PSC	​	reference	​	​	reference	​
TSC	2.858	1.742–4.689	<0.001	2.914	1.744–4.867	<0.001
Detection-to-door time	1.000	0.990–1.009	0.955	​	​	​
Onset-to-door time	1.000	0.999–1.000	0.194	​	​	​

MPSS, Maria Prehospital Stroke Scale; OR, odds ratio; CI, confidence interval; PSC, primary stroke center; TSC, thrombectomy-capable stroke center.

## Discussion

The AUROC for all four assessment tools in diagnosing stroke were comparable (approximately 0.7), with no statistically significant differences observed in pairwise comparisons (Table 4). These findings suggest that the inclusion of LVO-related items into the CPSS or MPSS does not enhance overall diagnostic accuracy for stroke. Moreover, multivariate logistic regression analysis identified only three prehospital assessment items – facial droop, arm drift, and dysarthria – as significantly associated with stroke diagnosis. Taken together, these results indicate that the standalone use of CPSS or MPSS may be appropriate for prehospital stroke screening.

In contrast, for LVO prediction, multivariate logistic regression analysis revealed that all assessment items except sex had statistically significant adjusted odds ratios. Although age was significant, its adjusted odds ratio was relatively low (1.023) compared to the other four items. Given the minimal contribution of age to predictive performance and the need to maintain simplicity in prehospital scales, age was excluded from the LVO assessment tool. Instead, we adopted the four items recommended by the Japan Stroke Society – aphasia, USN, conjugate eye deviation, and arrhythmia – as components of the LVO evaluation scale. Unlike stroke prediction, pairwise comparisons of ROC curves demonstrated that the addition of these four LVO-related items significantly improved the predictive performance of both MPSS and CPSS (*p* < 0.001 for MPSS; *p* < 0.001 for CPSS; Table 4B). Among the four tools, the MPSS+4 scale showed the highest predictive capacity and was significantly superior to the CPSS+4 scale (*p* = 0.029). Notably, the standalone CPSS failed to exceed an AUROC of 0.7 for either stroke or LVO diagnosis, indicating limited discriminatory ability. While balancing simplicity and diagnostic accuracy in prehospital LVO triage remains a challenge, our study raises questions about whether CPSS alone can fully capture both stroke and LVO in the prehospital setting, highlighting the need for additional research.

Furthermore, the MPSS+4 scale demonstrated a positive correlation with the likelihood of patients receiving MT after hospital arrival (Table 6), underscoring its potential utility as a novel and practical tool for EMTs to anticipate MT candidacy in the field. Based on these results, the Kawasaki City Medical Control Council has decided to adopt a new transfer protocol in which stroke diagnosis is first performed using the MPSS, followed by LVO assessment using the MPSS+4 scale if stroke is suspected. The Council has initiated efforts to evaluate the triage effectiveness of this revised protocol.

We defined LVO as occlusion of both the anterior circulation (internal carotid artery, anterior cerebral artery, and middle cerebral artery including M1 and M2 segments) and the posterior circulation (basilar artery). This definition is consistent with *the Japan Stroke Society Guideline 2021 for the Treatment of Stroke* [24], which outlines eligibility criteria for endovascular therapy. In contrast, many previous studies have limited the definition of LVO to anterior-circulation occlusions [25]. When we recalculated the predictive performance of the MPSS+4 scale after excluding basilar artery occlusion from the LVO definition, the AUROC was 0.775, which was similar to the AUROC obtained when basilar artery occlusion was included (0.768). These findings suggest that the robustness of the MPSS+4 scale is not compromised by differences in the LVO definition.

In this study, we adopted the aphasia and USN assessment methods used in the ELVO screen reported by Suzuki et al. [26]. They reported frequencies of 35% for aphasia and 25% for USN among patients with suspected stroke, whereas the corresponding frequencies in our study (18.7% and 5.7%, respectively) were considerably lower. In their study, physicians at stroke centers instructed EMTs by telephone on how to assess conjugate eye deviation, aphasia, and neglect and recorded the results for LVO prediction. In contrast, our study included all stroke-related EMS transports within a single metropolitan area, and all EMTs underwent video-based training – including distinctions between aphasia and impaired consciousness and differentiation of USN – before performing onsite assessments independently. This discrepancy in detection frequencies does not indicate that our EMTs failed to correctly detect cortical signs in stroke patients; rather, it is likely attributable to differences in study design.

This study successfully elucidated the clinimetric properties of the MPSS+4 scale; however, further investigation is warranted to determine whether the implementation of new transport protocols contributes to improved stroke outcomes across entire healthcare regions. In the RACECAT trial [27], no significant difference in 3-month outcomes was observed between patients suspected of stroke who were directly transported to TSCs using the RACE scale [28] and those transported to the nearest PSCs. For false-positive cases identified as LVO by the RACE scale, long-distance transport may result in clinical disadvantages. Notably, among 302 patients ultimately diagnosed with intracerebral hemorrhage in the RACECAT study, those transported directly to TSCs (mothership group) had significantly worse 90-day mRS scores compared to those in the standard transport group. These findings underscore the importance of geographical factors – such as the distribution and proximity of TSCs and PSCs – when evaluating the impact of LVO transport protocols [29]. Therefore, it is advisable that regional policymakers determine transport strategies based on the sensitivity and specificity of the selected LVO scale, and engage in ongoing post-implementation evaluation to develop triage methods tailored to the characteristics of each region.

A major strength of this study lies in its use of real-world data, specifically the findings assessed EMTs at the scene prior to hospital transport. Additionally, the analysis was based on a comprehensive survey of all stroke transports within the city, enhancing its representativeness. However, several limitations should be acknowledged. Although numerous LVO prediction scales have been reported to date, this study evaluated only four, precluding direct comparison with other scales. A recent study comparing 14 anterior-circulation LVO (aLVO) scales using 2,358 transport cases reported AUROC values of 0.81 for the Rapid Arterial Occlusion Evaluation (RACE), 0.80 for the Los Angeles Motor Scale (LAMS), 0.80 for the Gaze-Face-Arm-Speech-Time (G-FAST), and 0.79 for the modified G-FAST (mG-FAST). While direct comparison with these studies is not feasible, the AUROC of the MPSS+4 scale in our study was 0.791, indicating an acceptable level of discriminatory capacity.

## Acknowledgments

The authors wish to thank Mr. Yusaburo Sasaki, Mr. Hiroshi Nishiyama (Kawasaki City Fire Department), Mr. Masaaki Kanda, Mr. Takahiro Watanabe (Health and Welfare Bureau, Kawasaki City), and Ms. Yoshiko Miyake for their continuous work for the citywide quality assurance monitoring. The Kawasaki Stroke Network Investigators include: Takahiro Shimizu, Yasuhiro Hasegawa, Yoshifumi Tsuboi, Toshihiro Ueda, Jinichi Sasanuma, Atsushi Tsuchiya, Ririko Takeda, Yohichi Imaizumi, Hajime Ono, Satoshi Inoue, Hiroyuki Nozaki, Takehiko Nagao, Hajime Nakanishi, Shojiro Kawaguchi, Kengo Yokomine, Hisanao Akiyama, and Yoshihisa Yamano.

## Statement of Ethics

This study was approved by the St. Marianna University Bioethics Committee (#2509). Following unlinkable anonymization, the dataset is subjected to biannual statistical analysis for quality assurance monitoring by the Department of Neurology at St. Marianna University School of Medicine, which serves as the administrative office of the Kawasaki Stroke Network. Due to the anonymous nature of the data, informed consent was waived. In accordance with these regulations, interventional studies or follow-up investigations beyond the acute phase were not permitted.

## Conflict of Interest Statement

The authors have no conflicts of interest to declare.

## Funding Sources

The present work was supported in part by a grant from the City of Kawasaki awarded to the Kawasaki Stroke Network (Principal Investigator: Yasuhiro Hasegawa, MD, PhD).

## Author Contributions

Takahiro Shimizu, Yasuhiro Hasegawa, Hajime Ono, Hisanao Akiyama, and Yoshihisa Yamano contributed to the conception and design of the study. Takahiro Shimizu and Yasuhiro Hasegawa drafted and revised the manuscript. Yasuhiro Hasegawa performed the data analysis, obtained ethical approval, and oversaw funding and supervision. Yoshifumi Tsuboi, Toshihiro Ueda, Jinichi Sasanuma, Atsushi Tsuchiya, Ririko Takeda, Yohichi Imaizumi, Satoshi Inoue, Hiroyuki Nozaki, Takehiko Nagao, Hajime Nakanishi, Shojiro Kawaguchi, and Kengo Yokomine contributed to data collection and interpretation. All authors approved the final version.

## Data Availability

The data that support the findings of this study are not publicly available due to requirements of the ethical approval received as they may contain information that could compromise the privacy of research participants but may be available in an anonymized format from the corresponding author.
